# A Survey of Surgical Management of Acute Cholecystitis in Eastern Saudi Arabia

**DOI:** 10.4103/1319-3767.54748

**Published:** 2009-07

**Authors:** Abdulmohsen A. Al-Mulhim

**Affiliations:** Department of Surgery, King Fahd Hospital of the University, Al-Khobar, Saudi Arabia

**Keywords:** Acute cholecystitis, hospital stay, laparoscopic cholecystectomy, management, survey

## Abstract

**Background/Aim:**

It is now 60 years since early cholecystectomy was advocated for acute cholecystitis (AC). Yet, surgical opinion remains divided regarding its optimal timing. Furthermore, recent surveys have shown low utilization of early laparoscopic cholecystectomy (LC) for AC.

**Aim:**

This survey aimed to assess the current management of AC in Eastern Saudi Arabia.

**Materials and Methods:**

A postal survey was conducted by means of a questionnaire sent to 95 surgeons practicing LC. The questionnaire addressed the surgical management of AC in relation to the subspecialty of interest, duration of consultant status, number of cholecystectomies performed per year, and the percentage performed laparoscopically.

**Results:**

There were 87 responders (92%); two were excluded from the analysis for different reasons. Early LC was preferred by 71% of the responders. With regard to the timing of LC, there was no significant difference in relation to the surgeon's subspecialty of interest or duration of consultant status. However, increased number of cholecystectomies and percentage of cholecystectomies performed with a laparoscopic approach were significantly associated with early LC.

**Conclusion:**

Early LC for AC is practiced by the majority of surgeons in Eastern Saudi Arabia. This practice is significantly associated with increased number of cholecystectomies performed as well as with the percentage performed with a laparoscopic approach. According to the current literature, early LC for AC results in a shorter total hospital stay and reduced cost of treatment.

Acute cholecystitis (AC) is the admission diagnosis in 11-50% of patients subjected to cholecystectomy.[1–3] In 27-51% of patients, AC can progress to serious complications such as gallbladder empyema, gangrene, perforation, or pericholecystic abscess.[2–6]

In 1948, Barksdale and Johnston conducted a survey among 151 members of the Southern Surgical Association (USA) on the management of AC. The majority (66.8%) of the responders favored early cholecystectomy. Accordingly, the association recommended urgent LC for AC.[7] Furthermore, before the introduction of laparoscopic cholecystectomy (LC), randomized controlled trials of early *vs* delayed open cholecystectomy showed that early surgery was associated with less blood loss, shorter operation time, fewer complications, shorter hospital stay, more rapid recovery, and reduced cost.[8–11]

However, the surgical consensus on early ‘open cholecystectomy’ for AC did not immediately apply to laparoscopic techniques. On the contrary, AC was initially considered a contraindication to LC[12] because of the high conversion rates to open cholecystectomy[13] and an unacceptable number of common bile duct injuries.[14] With increase in experience and improvements in instrumentation and technique, an increasing numbers of surgeons are opting for early LC for AC.

The results of randomized clinical trials have clearly shown that for AC, the laparoscopic approach is superior to open cholecystectomy as there is reduced morbidity, shorter hospital stay, more rapid recovery, and reduction in the overall cost of treatment.[4,15] Furthermore, compared with delayed LC, early LC for acute cholecystitis is safe and results in a lower rate of conversion to open cholecystectomy, shorter hospital stay, more rapid recovery, and reduced cost.[16–18] Early LC also avoids the problems associated with delayed intervention, including failure of initial medical treatment and need for readmission with recurrent complications before the interval cholecystectomy.[16,19]

Despite these convincing results, there is a wide variation in the use of early LC for AC. A nationwide study from the USA revealed that 80% of patients admitted with AC had early cholecystectomy;[20] in contrast, the corresponding figures from England and Japan are 15%[21] and 42%,[22] respectively.

To date, there have been only five surveys conducted on the surgical management of AC, including three from the UK.[22–26] To the best of the author's knowledge, there has been no previous survey in Saudi Arabia. This paper reports the current surgical management of uncomplicated acute gallstone cholecystitis in the Eastern Province of Saudi Arabia.

## MATERIALS AND METHODS

The names and addresses of the practicing consultant general surgeons were obtained from the office of the Saudi Commission for Health Specialties in the Eastern Province of Saudi Arabia. This list was cross-checked with that of the Surgical Club in the same region. In January 2008, a questionnaire was posted to 95 surgeons who practice LC. The questionnaire addressed the surgeon's management of AC [Appendix]. Responses were anonymous. All replies were received by mid-March 2008. Data were collected and checked by the author. The Chi square test was used to compare the differences between two groups; *P* < 0.05 was taken to indicate statistical significance.

## RESULTS

There were 87 replies, i.e., a response rate of 91.6%. Two of the responses were excluded from the analysis: One because the concerned surgeon was not practicing LC and the other because the data provided was incomplete. All the remaining 85 responding surgeons were routinely performing LC. Overall, 60 (70.6%) surgeons preferred early LC for AC.

### Timing of cholecystectomy in relation to subspecialty of interest

Of the 85 responding surgeons, 55 (64.7%) had upper gastrointestinal (GI) and hepato-pancreato-biliary (HPB) interests. As shown in Table 1, 76.4% of surgeons with upper GI/HPB interests opted for early surgery *vs* 63.3% of those with other interests (*P* = 0.2).

**Table 1:** Surgical management of uncomplicated acute cholecystitis

Relative to	Early cholecystectomy *n* (%)	Delayed cholecystectomy *n* (%)	Total	*P* value
*Specialty of interest				
Upper GI/HPB	42 (76.4)	13 (23.6)	55	0.2
Others	19 (63.3)	11 (36.7)	30	
†Years as consultant				
(A) 1-10	22 (66.7)	11 (33.3)	33	A:B = 0.9
(B) 11-20	23 (67.6)	11 (32.4)	34	B:C = 0.09
(C) > 20	16 (88.9)	2 (11.1)	18	A:C = 0.08
‡No. of cholecystectomies/year				
0-50	25 (59.5)	17 (40.5)	42	0.01
>50	36 (83.7)	7 (16.3)	43	
§Percentage of LC				
≤90	4 (36)	7 (64)	11	0.005
>90	57 (77)	17 (23)	74	

GI = gastrointestinal; HPB = hepato-pancreato-biliary; others = general surgery, colorectal, breast, endocrine, pediatric; LC = laparoscopic cholecystectomy. Percentages are of the total for that

* specialty of interest

† years as consultant

‡ number of cholecystectomies per year, and

§ percentage of LC

### Timing of cholecystectomy in relation to the length of experience as a consultant

A total of 52 (61%) of the responders had been consultants for >10 years. Almost 89% of senior surgeons (i.e., those with experience of >20 years) opted for early LC compared with 67% of those with experience of ≤10 years and 68% of those with experience of 11-20 years. The difference between the senior surgeons (i.e., those with >20 years experience) and the other two groups indicates a trend but fails to achieve statistical significance [Table 1].

### Timing of cholecystectomy in relation to number of cholecystectomies performed per year

Over 50% of the responders performed more than 50 cholecystectomies per year [Table 1]. There was a significant difference (*P* = 0.01) relative to the number of procedures done by the surgeon; 83.7% of surgeons who performed >50 cholecystectomies per year opted for early surgery, compared to 59.5% of those who performed ≤50 cholecystectomies per year.

### Timing of cholecystectomy in relation to percentage of LCs

The majority of surgeons (74; 87%) used the laparoscopic approach in over 90% of the cases; 77% of these surgeons opted for early surgery compared to 36% of those who used laparoscopy in ≤90% of their cases [Table 1]. The difference between these two groups was statistically significant (*P* = 0.005).

### Intraoperative cholangiography during emergency LC

The survey also looked at some subordinate information on intraoperative cholangiography (IOC). None of the surgeons routinely performed IOC during emergency LC; 32% of the surgeons performed IOC in selected cases, and the rest (68%) rarely performed this procedure.

## DISCUSSION

Since its introduction in our region, LC has rapidly replaced open cholecystectomy as the treatment of choice for gallstone disease.[27] Previous reports from our institution have indicated that early LC for AC is safe, with a low rate of conversion to open cholecystectomy, few complications, and no mortality.[1,28]

At present there is no specific protocol for the management of AC in Saudi Arabia. However, the satisfactory response rate of 92% documented here is a reflection of the enthusiasm of surgeons in Eastern Saudi Arabia towards AC. Although the survey in Queensland had a response rate of 92.7%,[23] other surveys only achieved response rates of between 54% and 72.5%.[22,24–26]

Almost 65% of surgeons in this study had upper GI/HPB interests; however, with regard to early or delayed LC, there was no significant difference between these surgeons and those with other interests (*P* = 0.2). Senapati *et al*. showed that, as expected, surgeons who had upper GI/HPB interests were significantly more likely to opt for a policy of early cholecystectomy.[24] Likewise, Campbell *et al*. showed that surgeons with upper GI or vascular interest favored early LC more frequently than those with interest in other subspecialties.[26]

Although early cholecystectomy was associated with increased experience, there were no significant differences based on the duration of consultant status [Table 1]. These findings may be explained by the fact that LC is a commonly performed procedure in all health services, and young surgeons become familiar with its technique sooner than with that of other major operations.

As expected, an increased number of cholecystectomies performed per year (>50) was associated with a significant increase in the use of early cholecystectomy (*P* = 0.01) [Table 1]. In addition, there was significant difference according to the percentage of cholecystectomies performed laparoscopically (<90% *vs* >90%; *P* = 0.005) [Table 1].

In contrast to the findings of other surveys,[22,23] a greater proportion of responders (68%) reported that they rarely performed IOC during emergency LC for AC. This finding is consistent with our belief that IOC should not be used ‘routinely’ during early LC for AC. This policy was not associated with increased rates of common bile duct injury or conversion rate.[1,28] Currently, there is robust evidence supporting selective use of IOC during emergency LC.[29,30]

Overall, LC was the technique preferred by 92% of the surgeons in our region, and 72% of all surgeons opted for early intervention. Despite the benefits of this policy, there seems to be no conclusive evidence regarding the optimal timing of early LC for AC. Most of the studies suggest that early LC for AC is best performed within 72-96 h of admission or within 7 days of onset of symptoms.[19,30] Others have advocated immediate LC within 24 h of presentation.[31] This approach, however, may not be feasible in every center, particularly for patients with complicated AC or comorbidities. Nevertheless, the majority of surgeons have shown that early LC for AC can be performed safely at any time during the index admission, with no increase in the conversion rate, morbidity, or duration of hospital stay.[32–36]

We agree with those who believe that LC for AC should be performed as early as possible, preferably within the first 72 h of admission and that a delay of more than 7 days can lead to increased risk of complications and conversion to open cholecystectomy, thus negating the benefits of laparoscopic surgery.[19,30,33]

It is not clear why there is a difference between our results and those of other surveys [Table 2]. Although no survey has quantified this issue, it is evident that a policy of delayed LC is mainly adopted due to limited resources, for example, a busy operating room, inadequate equipment, and lack of an experienced laparoscopic team, particularly outside of the regular working hours.[22–26] Other patient-related reasons include delay in presentation, lack of health insurance, and the presence of comorbidities that need to be managed before surgical intervention can be undertaken.[36,37]

**Table 2:** Review of current surveys on the surgical management of acute cholecystitis

Author, Ref. (country, year)	Response rate (%)	Analyzed responders (*n*)	Early cholecystectomy (%)	Delayed cholecystectomy (%)
Senapati *et al*.,[24] (UK, 2003)	54	515	20	80
Cameron *et al*.,[25] (UK, 2004)	72	308	11	89
Askew,[23] (Australia, 2005)	93	107	52	48
Yamashita *et al*.,[22] (Japan, 2006)	73	211	42	42
Campbell *et al*.,[26] (Scotland, 2007)	71	67	60	34
Al-Mulhim (Saudi Arabia, current study, 2009)	92	85	71	29

Our experience is somewhat different. Although the healthcare policy in this country is increasingly becoming insurance-led, access to health services is still available to all emergency patients regardless of their insurance status. In addition, our patients are younger than their counterparts in the West.[27] Medical comorbidities and severe AC preclude early intervention in elderly patients.[2,37] It is notable, that most of the previous surveys[22–25] were conducted and published before the release of the guidelines of the European Association for Endoscopic Surgery on laparoscopy for abdominal emergencies[38] and the Tokyo guidelines on surgical treatment of patients with acute cholecystitis.[39] Both guidelines advocate early LC in otherwise fit patients with AC.

A recent meta-analysis of randomized clinical trials comparing early *vs* delayed LC for AC showed that early surgery results in a significantly shorter total hospital stay at the cost of a significantly longer operation time, with no significant differences in conversion rates or complications.[30] Taking into consideration, for example, that between 600000-700000 cholecystectomies are performed per year in the United States alone,[37,40] a reduction of hospital stay even by one day would result in a significant reduction in overall hospital cost.

## CONCLUSION

Although limited in scope, this survey has been useful in determining the current regional practice. It can be the stepping stone to much bigger collaborative national studies. Such studies would promote a more measured change in overall practice, including the management of AC. In addition, provision of quality health services continues to expand in Saudi Arabia. Awareness of the trend documented here should be of general interest.

## APPENDIX

**A survey of the surgical management of uncomplicated acute cholecystitis in Eastern saudi Arabia**

1. What is your major subspecialty of interest?
  □ Upper GI
  □ Hepato-pancreato-biliary
  □ Others (specify)
2. Years in post as consultant?
  □ 1-10
  □ 11-20
  □ > 20
3. Do you regularly perform elective laparoscopic cholecystectomy?
  □ Yes
  □ No
4. How many laparoscopic cholecystectomies do you perform per year?
  □ 0-50
  □ >50
5. Which of the following management options do you usually adopt?
  □ Early laparoscopic cholecystectomy (during same admission)
  □ Conservative treatment followed by delayed laparoscopic cholecystectomy at a later admission
6. Do you usually perform intraoperative cholangiography during urgent cholecystectomy?
  □ Rarely
  □ Selectively
  □ Almost always
